# Risk factors for negative blood cultures in adult medical inpatients – a retrospective analysis

**DOI:** 10.1186/1471-2334-8-148

**Published:** 2008-10-28

**Authors:** Boris P Ehrenstein, Vera Ehrenstein, Christine Henke, Hans-Jörg Linde, Bernd Salzberger, Jürgen Schölmerich, Thomas Glück

**Affiliations:** 1 Dept. of Internal Medicine (I), University of Regensburg, Germany; 2 Dept. of Epidemiology, School of Public Health, Boston University, USA; 3 Dept. of Medical Microbiology and Hygiene, University of Regensburg, Germany

## Abstract

**Background:**

The identification of clinical factors associated with negative blood cultures could help to avoid unnecessary blood cultures. C-reactive protein (CRP) is a well-established inflammation marker commonly used in the management of medical inpatients.

**Methods:**

We studied the association of clinical factors, CRP levels and changes of CRP documented prior to blood culture draws with the absence of bacteremia for hospitalized medical patients.

**Results:**

In the retrospective analysis of 710 blood cultures obtained from 310 medical inpatients of non-intensive-care wards during one year (admission blood cultures obtained in the emergency room were excluded), the following retrospectively available factors were the only independent predictors of blood cultures negative for obligate pathogens: a good clinical condition represented by the lowest of three general nursing categories (OR 4.2, 95% CI 1.8 – 9.5), a CRP rise > 50 mg/L documented before the blood culture draw (OR 2.0 95% CI 1.8–9.5) and any antibiotic treatment in the previous seven days (OR 2.0, 95% CI 1.1–3.5).

**Conclusion:**

Including the general clinical condition, antibiotic pre-treatment and a substantial rise of CRP into the decision, whether or not to obtain blood cultures from medical inpatients with a suspected infection, could improve the diagnostic yield.

## Background

Blood cultures (BC) are especially important in the diagnosis of endovascular infections. At the same time, the benefits and cost effectiveness of blood culture diagnosis for many other infectious syndromes are less clearly defined. The identification of clinical factors associated with negative blood cultures could help to avoid unnecessary BC. Serum C-reactive protein (CRP) is a well-established marker of inflammation [1,2]. It is commonly used in many clinical settings to evaluate and monitor severity of disease in patients with presumed infections [1]. We aimed to evaluate associations of different CRP levels or changes in CRP prior to BC draw with subsequent BC results. The association of negative BC results with other clinical factors commonly used to decide whether a BC draw is indicated and available by chart review were studied additionally.

## Methods

At our tertiary care university hospital, we reviewed medical charts of all 363 patients from three general internal medicine non-intensive-care wards with at least one BC draw in the year 2000 (blood volume per BC app. 15 – 20 mL, split and cultivated in an aerobic and an anaerobic bottle, BacT/Alert™, Organon Teknika, Durham, NC). During the study period, no formal guidelines for the indication of BC existed in our department. Ordering BC was therefore solely based on the judgement of the treating physicians.

Clinical relevance of skin flora isolated in BC (coagulase-negative staphylococci, *Corrynebacterium *spp., and *Propionibacterium *spp.) is difficult to determine, even prospectively. Therefore in our retrospective study, these bacterial species were regarded as either contaminations or the cause of endovascular catheter infections and were excluded from the analysis. All other isolated pathogens were considered to be obligate pathogens (OP).

We retrieved the following variables – all of which had been available to treating physicians at the time point of BC-collection – and evaluated their association with BC results: BC draw in the emergency room (ER) or intensive care unit (ICU) in the previous 3 days; any antibiotic treatment in the previous 7 days; any intravenous antibiotics in previous 2 days; lack of high fever (temperature of < 40°C); absence of documented substantial temperature rise (no rise or rise of < 2°C); absence of white blood cell count (WBC) elevation (value of < 12/nL); absence of a documented substantial WBC increase (no rise or rise < 2/nL); substantial (value of > 100 mg/L) elevation of CRP level; documented substantial rise of CRP (rise of > 50 mg/L); lowest general nursing category (reflecting dependence on help with daily activities, 1 vs. 2 or 3, as surrogate markers for the patients' overall clinical condition); age below 60 years. Only observations documented within 24 hours before the BC draw (within 48 h for WBC and CRP values) were included in the analysis. All factors reflecting changes over time were determined by the subtraction of the two most recent values obtained before the BC draw which were at least 24 hours apart. Additionally the main hospital discharge diagnosis of all patients with BC draws was obtained.

Crude analyses were performed using χ^2^-test, and adjusted analyses, using logistic regression, with the final predictive model derived by a backward elimination (exclusion criterion, p > 0.05). All analyses were performed using SAS software (SAS Institute Inc. Cary, NC).

The research was conducted in accordance with requirements set for retrospective studies by the local ethics committee (ethics committee of the University of Regensburg, Germany).

## Results

A total of 856 BC from 363 patients on three analyzed non-ICU wards in the year 2000 were identified. 710 (83%) of BC of 310 (85%) patients had sufficient clinical documentation to be included in the analysis. 96 (13%) of 710 BC were positive, and in 91 the species could be identified. Thirty-two results were regarded as contamination or as related to endovascular catheter infection (coagulase-negative staphylococci (n = 30), *Corrynebacterium *spp. (n = 1), *Propionibacterium *acnes (n = 1)), and remaining 59 as obligate pathogens (OP): Gram-negative bacteria n = 35; Gram-positive bacteria n = 16; *Candida *spp. n = 8). Comprising blood cultures drawn within 3 days as one diagnostic episode (DE), there were 426 DE, 41 (10%) of which yielded OP. Of the 157 DE with more than 1 BC, 17 (11%) DE yielded OP. An analysis stratified by the amount of BC per DE (1–2 vs. 3 or more BC per DE) did not reveal a significant difference in the rate of OP-positive results (35/445 (7.9%) vs. 24/265 (9.1%) p = 0.57 by χ^2^-test). Furthermore, an analysis stratified by hospital day (BC obtained on day 1 and 2 vs. > day 2 of the hospital stay) showed no significant difference in the rate of OP-positve BC: 25/330 (7.6%) vs. 34/380 (8.9%); (p = 0.51 by χ^2^-test).

There were no significant differences (χ^2^-test for 5 strata, p = 0.63) in the crude rates of OP-positive BC across strata of CRP values determined prior to drawing the BC: 0 – 5 mg/L (normal range) 2/20 (10.0%), 6–50 mg/L 15/196 (7.7%), 51–100 mg/L 14/133 (10.6%), 101–200 mg/L 14/219 (6.4%), and CRP > 200 mg/L 14/141 (9.9%). There was a significant difference (χ^2^-test for 4 strata, p = 0.008) in the crude rates of OP-positive BC among strata of the documented change of CRP prior to drawing the BC: any fall in CRP 11/207 (5.3%), CRP rise between 1 and 50 mg/L 31/261 (11.9%), CRP rise > 50 mg/L 4/102 (3.9%), and change not applicable (only one CRP value determined prior to drawing the BC) 13/140 (9.3%). This difference remained statistically significant analyzing only the subgroup of 291BC from patients with no antibiotic therapy in the previous 7 days. For the subgroup of 61 BC from patients with any antibiotic therapy in the previous 7 days and a substantial rise of CRP (> 50 mg/L) prior to drawing the BC, the number of BC needed to diagnose one OP was 20 (61/3).

A summary of crude and adjusted analyses is shown in Additional file 1. In the crude analysis, the following factors were significantly associated with OP-negative BC: BC draw in the ER or ICU in the previous 3 days, absence of high fever, absence of a documented substantial temperature rise, absence of a documented substantial rise of the WBC, and the lowest general nursing category. After considering all factors simultaneously in the logistic regression model (c-statistic 0.665; Hosmer-Lemeshow goodness-of-fit statistic 0.612; discordant prediction 20.4%), low general nursing category (odds ratio (OR) 4.2), a CRP rise > 50 mg/L (OR 2.0), and any antibiotic in the previous 7 days (OR 2.0) remained significantly (p < 0.05) associated with OP-negative BC.

Analyzing the main hospital discharge diagnosis, 23/332 (6.9%) BC drawn from patients with a non-infectious and 36/458 (7.9%) BC drawn from patients with an infectious main hospital discharge diagnosis were OP-positive. Among BC obtained from patients with infectious main hospital discharge diagnosis, OP-positive rates were as follows: abdominal infections 24/154 (15.6%); soft tissue infections 1/58 (1.7%); respiratory infections 1/86 (1.2%); sepsis 8/47 (17.0%); catheter infections 0/10 (0%); urogenital infections 0/9 (0%); infections in HIV-positive patients 0/9 (0%); endocarditis 2/2 (100%); meningitis 0/3 (0%).

## Discussion

Several major studies have identified clinical factors and derived clinical prediction models for positive BC results [3-5]. These studies did not assess the role of CRP. We evaluated the association of CRP values and CRP change prior to obtaining BC with OP-negative BC results in adult internal medicine non-ICU patients and compared this association with that of selected other factors, commonly used in the decision on ordering BC available by chart review.

The level of CRP available before drawing the BC showed no association with BC results in our analysis. In accordance, a study of CRP values on the day of BC draw in patients with bacteremia showed no significant difference for CRP levels between OP-positive BC and contamination-positive BC [6]. Several studies, assessing the usefulness of CRP measured at the onset of fever in adult neutropenic cancer patients, showed no significant association [7,8] or only a modest association with the presence of bacteremia [9]. In a retrospective analysis of adult inpatients with sepsis syndrome without shock, there was no significant difference of CRP values between bacteremic and non-bacteremic patients at the time of obtaining the BC [10]. Contrary to these studies, Tokuda et al. found in a population with a low prevalence (4%) of malignant conditions a significant difference of CRP values between bacteremic and non-bacteremic ER patients with suspected infections [11]. They therefore included the variable of CRP < 100 mg/L in a classification algorithm for low risk of bacteremia.

There was an association between a substantial (> 50 mg/L) rise in CRP determined prior to BC draw and OP-negative BC in this study. To our knowledge, so far no study has assessed changes of CRP prior to BC draw as a predictor for bacteremia among general internal medicine adult inpatients. At a first glance, it seems counterintuitive that a rise of an inflammation marker is associated with low prevalence of bacteremia. We speculate that this finding could be explained by the time-dependent course of CRP level after an inflammatory stimulus. Rintala and co-workers showed that CRP levels peak between 24 and 48 h after admission for an infection [2,12]. Therefore, a substantial rise of CRP could indicate diagnostic situations, where the inflammatory stimulus – e.g. transient bacteremia – may already have disappeared by the time the BC is drawn. This hypothesis is supported by our finding of a low prevalence of OP-positive BC not only with a prior rise but also obtained after having passed a peak of CRP values prior to obtaining BC.

A good overall clinical condition of the patient represented by the lowest of the 3 general nursing categories had the strongest association with OP-negative BC. We did not to evaluate disease-specific anamnestic information in our retrospective chart-based analysis. Therefore, the strong association of the overall clinical condition with BC results in the regression model was not surprising.

There are some limitations to our study. The retrospective design excluded the evaluation of certain anamnestic information and physical findings as competing clinical predictors. By design, we excluded BC from patients treated in the ICU. Therefore, our results might not apply to critical ill patients with a very high probability of bacteremia. The measurement of CRP was not performed at exactly specified time-points in relation to the obtaining of the BC. During the period studied, the measurement of procalcitonin as an inflammatory marker was clinically not available at our institution; therefore we could not evaluate the association of this promising new inflammatory marker with BC results in our retrospective cohort [13]. Because of the low frequency of positive BC, we refrained from splitting our cohort in a derivation and a validation sample. Many (46.8%) BC in our study were obtained from patients where the infection or presumed infection was not the main hospital discharge diagnosis. Therefore, our results should be confirmed in other clinical settings and especially the change of CRP prior to obtaining BC should be included in further prospective studies to learn more about the generalisability of our findings.

## Conclusion

The low rate of positive BC obtained from adult non-ICU inpatients with a passed or recent peak in CRP could be useful in the decision whether to draw or not to draw a blood culture. Recent changes in CRP or other inflammatory markers should be evaluated prospectively as candidate factors in future decision rules. Including the general clinical condition, antibiotic pre-treatment and a substantial rise of CRP into the decision, whether or not to obtain blood cultures from medical inpatients with a suspected infection, has the potential to improve the diagnostic yield of BC by refraining from BC-collection in situations with low positive-rates and therefore might lower costs.

## Competing interests

The authors declare that they have no competing interests.

## Authors' contributions

BPE designed the study, validated the chart review data, analyzed the data and wrote the manuscript, VE analyzed the data and revised the manuscript, CH performed the chart review and analyzed the data, HJL provided the microbiology data and revised the manuscript, BS, JS and TG designed the study and revised the manuscript.

## Pre-publication history

The pre-publication history for this paper can be accessed here:



## Supplementary Material

Additional file 1 **Table 1 – Crude and adjusted analyses of the association of clinical factors with BC negative for obligate pathogens**. A Microsoft Word document containing Table 1 of the manuscript. Click here for file
