# Supplementary material for: Risk factors for negative blood cultures in adult medical inpatients – a retrospective analysis
Source: BMC Infect Dis. 2008 Oct 28;8:148. doi: 10.1186/1471-2334-8-148 (PMC2582035; doi:10.1186/1471-2334-8-148)
Supplement: Additional file 1 — Table 1 – Crude and adjusted analyses of the association of clinical factors with BC negative for obligate pathogens. A Microsoft Word document containing Table 1 of the manuscript. [file 1471-2334-8-148-S1.doc]

# Tables

## Table 1 - Crude and adjusted analyses of the association of clinical factors with BC negative for obligate pathogens.

| **Clinical Factors** |  | | **Crude analysis** | | | |  |  | **Adjusted analysis^a^** | |
| --- | --- | --- | --- | --- | --- | --- | --- | --- | --- | --- |
|  | Number of BC with factor present | Percent negative for OP | | NND^b^ | Odds ratio | 95% CI | p^c^ |  | Odds ratio | 95% CI |
| Prior BC in ER or ICU in the last 3 days | 263 | 93.2 | | 15 | 1.4 | 0.8 - 2.4 | 0.27 |  |  |  |
| Any antibiotic in the previous 7 days | 419 | 92.8 | | 14 | 1.4 | 0.8 - 2.5 | 0.18 |  | 2.0 | 1.1 - 3.5 |
| Any intravenous antibiotic in the previous 2 days | 332 | 92.8 | | 14 | 1.3 | 0.8 - 2.3 | 0.33 |  |  |  |
| No high fever (temperature <40°C) | 597 | 92.6 | | 14 | 1.9 | 1.0 - 3.6 | 0.04 |  |  |  |
| No substantial rise in temperature (rise <2°C) | 576 | 92.9 | | 14 | 2.0 | 1.2 - 3.7 | 0.02 |  |  |  |
| No elevated WBC (value <12/nL) | 439 | 92.7 | | 14 | 1.4 | 0.8 - 2.4 | 0.21 |  |  |  |
| No substantial rise of WBC (rise <2/nL) | 556 | 93.0 | | 14 | 2.0 | 1.1 - 3.5 | 0.02 |  |  |  |
| Substantially elevated CRP level (value >100 mg/L) | 365 | 92.3 | | 13 | 1.2 | 0.7 - 2.0 | 0.53 |  |  |  |
| Substantial rise of CRP (rise > 50mg/L) | 102 | 96.1 | | 25 | 2.4 | 0.9 - 6.9 | 0.08 |  | 2.0 | 1.1 - 3.5 |
| Age below 60 years | 320 | 92.2 | | 13 | 1.1 | 0.7 - 1.9 | 0.66 |  |  |  |
| Lowest general nursing category (1 vs. 2 or 3) | 220 | 96.8 | | 31 | 3.6 | 1.6 - 8.1 | <0.01 |  | 4.2 | 1.8 - 9.5 |
| **All blood cultures** | **710** | **91.7** | | **12** | **-** | **-** | **-** |  | **-** | **-** |

Abbreviations: obligate pathogens (OP), blood culture (BC), number needed to diagnose (NND), confidence interval (CI), emergency room (ER), intensive care unit (ICU), white blood cell count (WBC), C-reactive protein (CRP)

^a^ Significant independent factors for logistic regression model were selected by stepwise backward procedure (SAS™ software)

^b^Number of BC needed to diagnose 1 OP in the specified subgroup

^c^ By χ^2^-statistic
